# Allergenic activity of *Pseudoterranova decipiens* (Nematoda: Anisakidae) in BALB/c mice

**DOI:** 10.1186/s13071-017-2231-4

**Published:** 2017-06-12

**Authors:** Alessandra Ludovisi, Gabriella Di Felice, Noelia Carballeda-Sangiao, Bianca Barletta, Cinzia Butteroni, Silvia Corinti, Gianluca Marucci, Miguel González-Muñoz, Edoardo Pozio, Maria Angeles Gómez-Morales

**Affiliations:** 1 Department of Infectious Diseases, Istituto Superiore di Sanità, viale Regina Elena 299, 00161 Rome, Italy; 2 University Hospital La Paz-FIBHULP, Paseo Castellana 261, 28046 Madrid, Spain

**Keywords:** Anisakidae, *Pseudoterranova decipiens*, *Anisakis pegreffii*, Sensitization, Allergic reactions, BALB/c mice, Immune response

## Abstract

**Background:**

*Anisakis simplex* is the only fishery-product associated parasite causing clinical allergic responses in humans so far. However, other anisakids, due to the presence of shared or own allergens, could also lead to allergic reactions after sensitization. The aim of this study was to determine if *Pseudoterranova decipiens* belonging to the family Anisakidae has allergenic activity and is able to induce sensitization after oral administration in a murine (BALB/c mice) model.

**Results:**

The ingestion of *A. pegreffii* proteins by BALB/c mice, which had been previously sensitized by intraperitoneal inoculation with the corresponding live L3 larvae, triggers signs of allergy within 60 min, whereas *P. decipiens* did to a lesser extent. Beside symptoms, allergic reactions were furtherly supported by the presence of histamine in sera of sensitized mice. Specific IgG1 and IgE responses were detected in sera of all sensitized mice from week four. Specific IgG2a response was detected in sera from mice sensitized to *P. decipiens*. After polyclonal or specific activation with anti-CD3/anti-CD28 or antigens, respectively, splenocytes from mice infected i.p. with *A. pegreffii* or *P. decipiens* larvae showed significantly higher production of IL-10 than naïve mice. After stimulation with specific antigens, significantly higher IL-5 and IL-13 amounts were produced by specific antigen stimulated splenocytes than by the naïve cells; only *P. decipiens* proteins induced IFN-ɣ.

**Conclusions:**

The overall results suggest that infection with *P. decipiens* can sensitize mice to react to subsequent oral challenge with anisakid proteins, as described for *A. simplex* (*sensu stricto*) and *A. pegreffii* infections. The results show that anisakid proteins induce a dominant Th2 response, although *P. decipiens* could also induce a mixed type 1/type 2 pattern.

## Background

The family Anisakidae includes marine nematodes, which use marine mammals as primary definitive hosts. The anisakid third-stage larvae (L3) infect fish, mollusks and crustaceans, and consequently, humans may become accidental hosts if they consume raw or undercooked fish [1]. The resulting disease, named anisakidosis, can induce from a mild to severe pathology. This infection is often associated with acute gastrointestinal symptoms such as abdominal pain, diarrhea, nausea, vomiting and in some cases, with allergic reactions [2–4]. The family Anisakidae includes at least 24 genera, of which the more studied are *Anisakis*, *Pseudoterranova* and *Contracaecum* [5]; species of these genera appear to have similar life-cycles, and to share some antigens [4] although their host species vary [6–8]. So far, *Anisakis simplex* (sensu *lato*), i.e. *Anisakis simplex* (sensu stricto), *Anisakis pegreffii* and *Anisakis berlandi* are the only fishery-product associated parasites causing clinical allergic responses recognized by EFSA [9]. In fact, *A. simplex* (*s.l.*) exposure may lead to clinical signs as urticaria, rhinitis, bronco-constriction, cough and/or gastrointestinal responses. Acute allergic reactions can also be seen, including anaphylactic shock [2, 4, 7, 10]. The potential for type I hypersensitivity responses in acute anisakiasis is indicated by the fact that serum anti-*A. simplex* IgE levels increase rapidly during the first few days, moreover lesions of a nature consistent with type I, type III and type IV hypersensitivity reactions have been found in guinea pigs and rabbits orally infected with *Anisakis* spp. [2]. Murine models of allergy to *A. simplex* (*s*.*l*.), that mimic the human *A. simplex* (*s.l.*) allergy, have been developed to study the specific aspects of anaphylaxis induced by this parasite [11–16]. However, the involvement in allergic responses of anisakid species different from *A. simplex* (*s*.*l*.) has been to our knowledge neglected [17]. The aim of the present study was to determine if *Pseudoterranova decipiens* belonging to the family Anisakidae, has allergenic activity and is able to induce sensitization.

## Methods

### Parasite isolation and identification

Third-stage larvae (L3) of *A. simplex* (*s*.*l*.) were collected from a silver scabbard fish (*Lepidopus caudatus*) fished in the Mediterranean Sea and L3 of *Pseudoterranova* sp. were collected from Atlantic cods (*Gadus morhua*) fished in the North Atlantic Ocean. L3 were extensively washed one by one in 2% acetic acid-phosphate buffered saline (PBS) and then each larva was cut into two parts, one part was kept in 90% ethanol for the molecular identification by PCR/RLPF [18], and the other part was frozen at -20 °C. L3 from the silver scabbard fish were identified as *A. pegreffii*, whereas L3 from the Atlantic cods were identified as *P. decipiens*. Crude worm extracts (CWE) were prepared from frozen L3. Briefly, 50 frozen L3 were suspended in 0.5 ml of PBS and then homogenized 5 times for 30 s and sonicated in ice 5 times for 60 s. The suspension was left overnight at 4 °C under magnetic stirring and further centrifuged at 1500× *g* and at 4 °C for 15 min. The protein content of the centrifuged supernatant was measured by the Bradford method (Quick Start™ Bradford, BIO-RAD, Hercules, CA, USA). Moreover, 140 and 80 additional live L3 assumed to be *A. pegreffii* and *P. decipiens,* respectively, on the basis of their morphology [19], host and geographical origins, were collected to be injected into BALB/c mice*.*

### Experimental BALB/c model

#### Infection and immunization

Forty-nine eight-week old female BALB/c mice (20 ± 2 g) were housed in the Animal Care Unit of the Istituto Superiore di Sanità (ISS), Rome, Italy. Experiments were carried out according to the European Directive 63/2010.

To determine the allergenic activity of *A. pegreffii* antigens, 20 mice were allocated into 4 groups of 5 mice each (Table 1). Mice from the first group (1Ap) were orally infected with 2 live *A. pegreffii* L3 using an oral dosing curved cannula for gavage (16G) at week 0, then re-infected with 2 live *A. pegreffii* L3 at week 8, and orally challenged with 5 mg/mouse (0.25 mg/mouse g) of CWE from *A. pegreffii* (ApCWE) in a total volume of 200 μl of PBS at week 12. Mice from the second group (2Ap) were anesthetized with 50 mg/kg ketamine (Ketavet®, Pfizer, Berlin, Germany) and 3 mg/kg xylazine (Rompun, Bayer Health Care, Germany); then, the abdominal skin was held and raised by a forceps and mice were injected intraperitoneally (i.p.) with 2 live *A. pegreffii* L3 at week 0, re-injected i.p. at week 8, and orally challenged with 5 mg/mouse of ApCWE at week 12. Mice from the third group (3Ap) were orally immunized with 700 μg/mouse of ApCWE at week 0 and week 8, and then orally challenged with 5 mg/mouse of ApCWE at week 12. Mice from the fourth group (4Ap) used as control, were orally inoculated with PBS following the same protocol as the other experimental groups (Table 1). Allergic reactions were evaluated after the oral challenge by the following scoring system: 0, no symptom; 1, scratching and rubbing around the nose and head, hypersensitivity to touch, irritability/aggression; 2, diarrhea, puffiness around the eyes and mouth, pilar erecti, reduced activity, and/or decreased activity with increased respiratory rate; 3, labored respiration, cyanosis around mouth and tail; 4, loss of consciousness; and 5, death [14].

**Table 1:** Experimental model of allergic sensitization and anaphylactic response in BALB/c mice with *Anisakis pegreffii* or *Pseudoterranova decipiens*

Mice group (5 mice per group)	Infection	Re-infection	Challenge	Evaluation of allergic reactions 60 min after the last challenge	Evaluation of the immune responses 24 h after the last challenge
*Anisakis pegreffii*					
1 Ap	2 L3 *per os*	2 L3 *per os*	5 mg CWE *per os*	yes	yes
2 Ap	2 L3 i.p.	2 L3 i.p.	5 mg CWE *per os*	yes	yes
	Inoculation	Challenge	Challenge		
3 Ap	700 μg CWE *per os*	700 μg CWE *per os*	5 mg CWE *per os*	yes	yes
4 Ap	PBS *per os*	PBS *per os*	PBS *per os*	yes	yes
Week p.i.	0	8	12	12	12
*Pseudoterranova decipiens*					
	Infection	Re-infection	Challenge		
1 Pd	2 L3 i.p.	2 L3 i.p.	5 mg CWE *per os*	yes	yes
	Inoculation	Challenge	Challenge		
2 Pd	700 μg CWE *per os*	700 μg CWE *per os*	5 mg CWE *per os*	yes	yes
3 Pd	PBS *per os*	PBS *per os*	PBS *per os*	yes	yes
Week p.i.	0	8	12	12	12

To determine the allergenic activity of *P. decipiens* antigens, 15 mice were allocated into 3 groups of 5 mice each (Table 1). Mice of the first group (1Pd) were anesthetized as above reported; then mice were injected i.p. with 2 live *P. decipiens* L3 at week 0, re-injected at week 8, and orally challenged with 5 mg/mouse of PdCWE at week 12. Mice of the second group (2Pd) were orally immunized on day 0 with 700 μg/mouse of PdCWE, and then re-inoculated with the same dose at week 8. At week 12, mice were orally challenged with 5 mg/mouse of PdCWE. Mice from the third group (3Pd) used as control, were orally inoculated with PBS following the same protocol that the other experimental groups (Table 1). Allergic reactions were evaluated after the oral challenge as above reported for *A. pegreffii*.

### Sera drawing and specific antibodies and histamine in serum

Blood samples were taken from each mouse by lateral tail vein bleeding at 0, 4, 8, 12 weeks and at 60 min after oral challenge. Individual sera were stored at -20 °C until analysis. Specific antibody responses (IgG1, IgG2a and IgE) were evaluated in individual serum samples by an in-house ELISA. Plates (Greiner Bio-One, Frickenhausen, Germany) were coated with CWE (5 μg/ml) from *A. pegreffii* or *P. decipiens*, and sera were diluted 1:20 (IgG2a and IgE) or 1:200 (IgG1). Results were expressed as Optical Density (OD). Histamine levels in the serum samples taken 60 min after the challenge [14], were measured by an enzyme immunoassay kit (IBL International, Hamburg, Germany).

### Cell cultures and cytokine levels in culture supernatants

The day after the evaluation of the allergic reactions, mice were sacrificed, and spleens were harvested under sterile conditions. After spleen disruption by a syringe and erythrocyte lysis, splenocytes were re-suspended in RPMI 1640 (Invitrogen Life Technologies, Gaithersburg, MD) supplemented with 10% FBS (Hyclone Laboratories, Logan, UT), 1 mM sodium pyruvate, 0.1 mM nonessential amino acids, 2 mM L-glutamine, 25 mM HEPES, 100 U/ml penicillin, 100 mg/ml streptomycin (all Invitrogen Life Technologies), and 0.05 mM 2-ME (Merck, Darmstadt, Germany) (complete medium) at a final concentration of 2.5 × 10^6^ cells/ml (24-well plates in duplicate, Costar Corporation, Cambridge, MA) for cytokine analysis. Cell cultures were stimulated with 50 μg/ml of CWE for 6 days or with anti-CD3 (10 μg/ml) and soluble anti-CD28 (2 μg/ml) monoclonal antibodies (BD Biosciences Pharmingen, San Josè, CA) for 3 days. At the end of the incubation period, supernatants were harvested for the cytokine (Interleukin (IL)-4, IL-5, IL-10 and Interferon (IFN)-ɣ) analysis and stored at -70 °C until assayed. The cytokine (IL-4, IL-5, IL-10 and IFN-ɣ) production was determined by commercial ELISA kits (eBioscience Affimetrix, San Diego, CA), according to the manufacturer’s instructions.

### Statistical analysis

The Mann-Whitney U-test was used to compare each experimental group with the relevant control group. *P*-value < 0.05 was considered significant.

## Results

### *Anisakis pegreffii* induces allergic symptoms in BALB/c mice, whereas *P. decipiens* does to a lesser extent

**Fig. 1:**
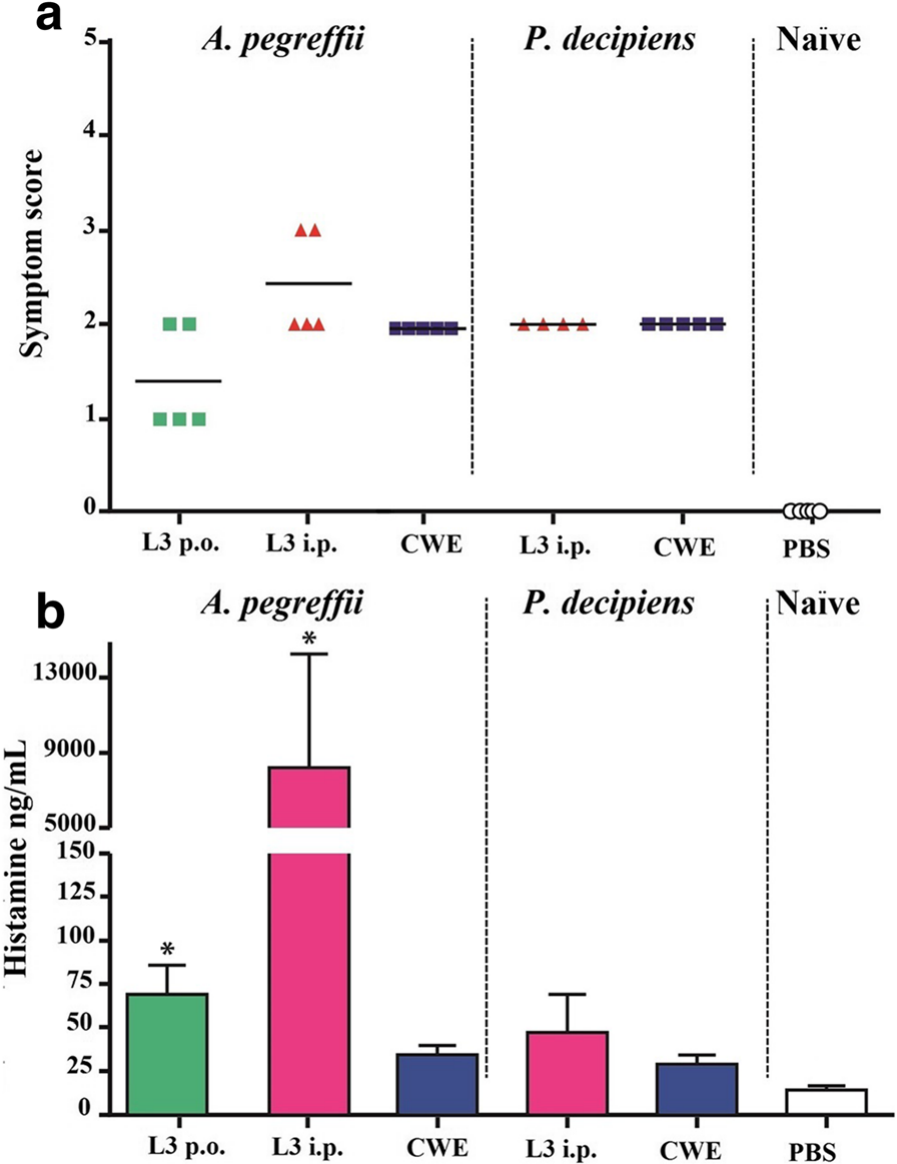
Allergic reactions in *Anisakis pegreffii* and *Pseudoterranova decipiens* sensitized mice. **a** Symptoms score in BALB/c mice. **b** Histamine release in BALB/c mouse sera. Green symbols and histograms: mice orally infected with *A. pegreffii* (L3 p.o.); red symbols and histograms: mice intraperitoneally infected with *A. pegreffii* or *P. decipiens* (L3 p.i.); blue symbols and histograms: mice orally immunized with *A. pegreffii* or *P. decipiens* crude worm extracts (CWE); open circles and histogram: mice (naïve) intraperitoneally inoculated with phosphate buffered saline (PBS). Symbols represent individual mice (combined results of 2–3 experiments). Horizontal bars represent medians. Reactions were scored following the protocol of Nieuwenhuizen et al. [14]. Histograms represent mean values with SE. Significances (*P < 0.05) were calculated between experimental and naïve control groups. Each experiment was conducted in triplicate

The gavage of *A. pegreffii* proteins to previously sensitized mice triggered signs of allergy within 60 min. The maximum symptom score (diarrhoea, reduced activity, and/or decreased activity with increased respiratory rate and cyanosis around the tail) was observed in animals injected i.p. with live L3. The ingestion of *P. decipiens* proteins in previously sensitized mice, induced irritability and reduced activity within 60 min, similarly to *A. pegreffii* (Fig. 1a). Differences in scoring among the *A. pegreffii* experimental groups were observed, whereas no difference was detected between the *P. decipiens* experimental groups. These results were further supported by the histamine presence in sera from all groups of mice inoculated with *A. pegreffii*, in which a higher histamine release than in the naïve control group (*P* < 0.05), was observed (Ap1*: U*_(0)_ = 2, *Z* = 2.50, *P* = 0.0118; Ap2*: U*_(__0)_ = 2, *Z* = 3.59, *P* = 0.0001). In *P. decipiens* sensitized mice, no significantly different histamine release was observed in comparison with the naïve control group (Fig. 1b). A certain variability among individual responses was observed in all groups of mice, despite inbred BALB/c mice were used.

### The i.p. infection of BALB/c mice with *A*. *pegreffii* L3 induces a dominant Th2 immune response, whereas *P. decipiens* induces a mixed Th1/Th2 response

**Fig. 2:**
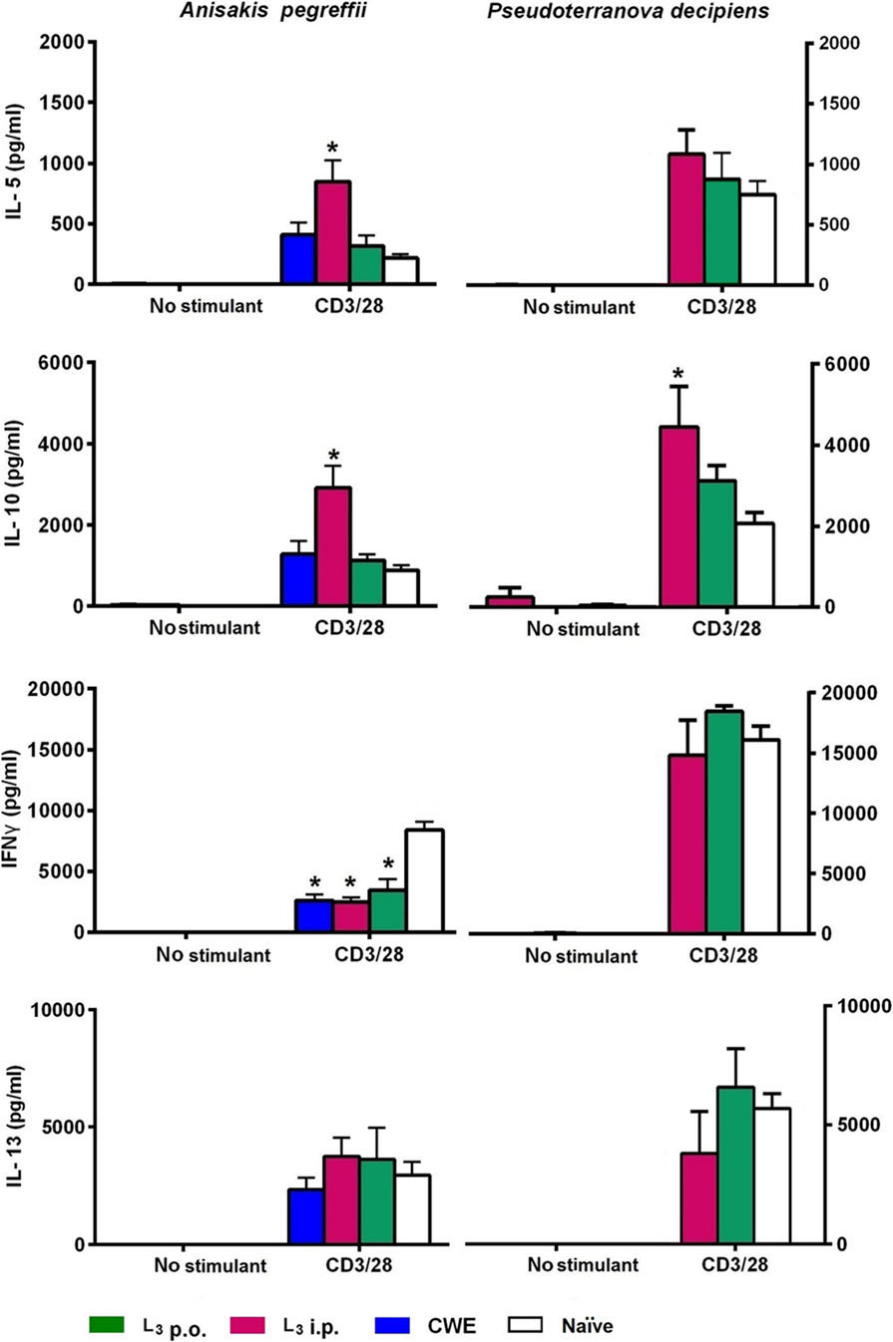
Cytokine production in supernatants of splenocyte cultures stimulated with anti-CD3 and anti-CD28 monoclonal antibodies at 10 μg/ml and 2 μg/ml, respectively. Splenocytes were harvested from *Anisakis pegreffii* or *Pseudoterranova decipiens* infected BALB/c mice at week 12 post - infection. Green histogram: mice orally infected with 2 *A. pegreffii* L3 (L3 p.o.); red histogram: mice intraperitoneally infected with 2 *A. pegreffii* L3 or 2 *P. decipiens* L3 (L3 p.i.)*;* blue histograms: mice orally inoculated with *A. pegreffii* or *P. decipiens* crude worm extracts (CWE); open histogram: mice intraperitoneally inoculated with phosphate buffered saline (PBS). Significances (*P < 0.05) were calculated between experimental and PBS control groups. Each experiment was conducted in triplicate

Polyclonal stimulation of splenocytes from mice inoculated i.p. with *A. pegreffii* L3 (group 2Ap) with anti-CD3/anti-CD28 induced significantly (*U*_(0)_ = 2, *Z* = 2.50, *P* = 0.01208) higher levels of IL-5 and IL-10 than in splenocytes from mice of 1Ap and 3Ap groups and naïve mice (Fig. 2). Splenocytes from mice inoculated i.p. with *P. decipiens* L3 (group 1Pd) produced IL-10 at a significantly (*U*_(0)_ = 2, *Z* = 2.50, *P* = 0.01208) higher level than that of naïve mice (Fig. 2). After activation with anti-CD3/anti-CD28, splenocytes from all *A. pegreffii* experimental mouse groups produced significantly (1Ap and Ap2: *U*_(0)_ = 2, *Z* = 2.50, *P* = 0.01208; Ap3 = *U*_(1)_ = 2, *Z* = 2.29, *P* = 0.02114) lower IFN-ɣ levels than those from the non-exposed naïve mice.

**Fig. 3:**
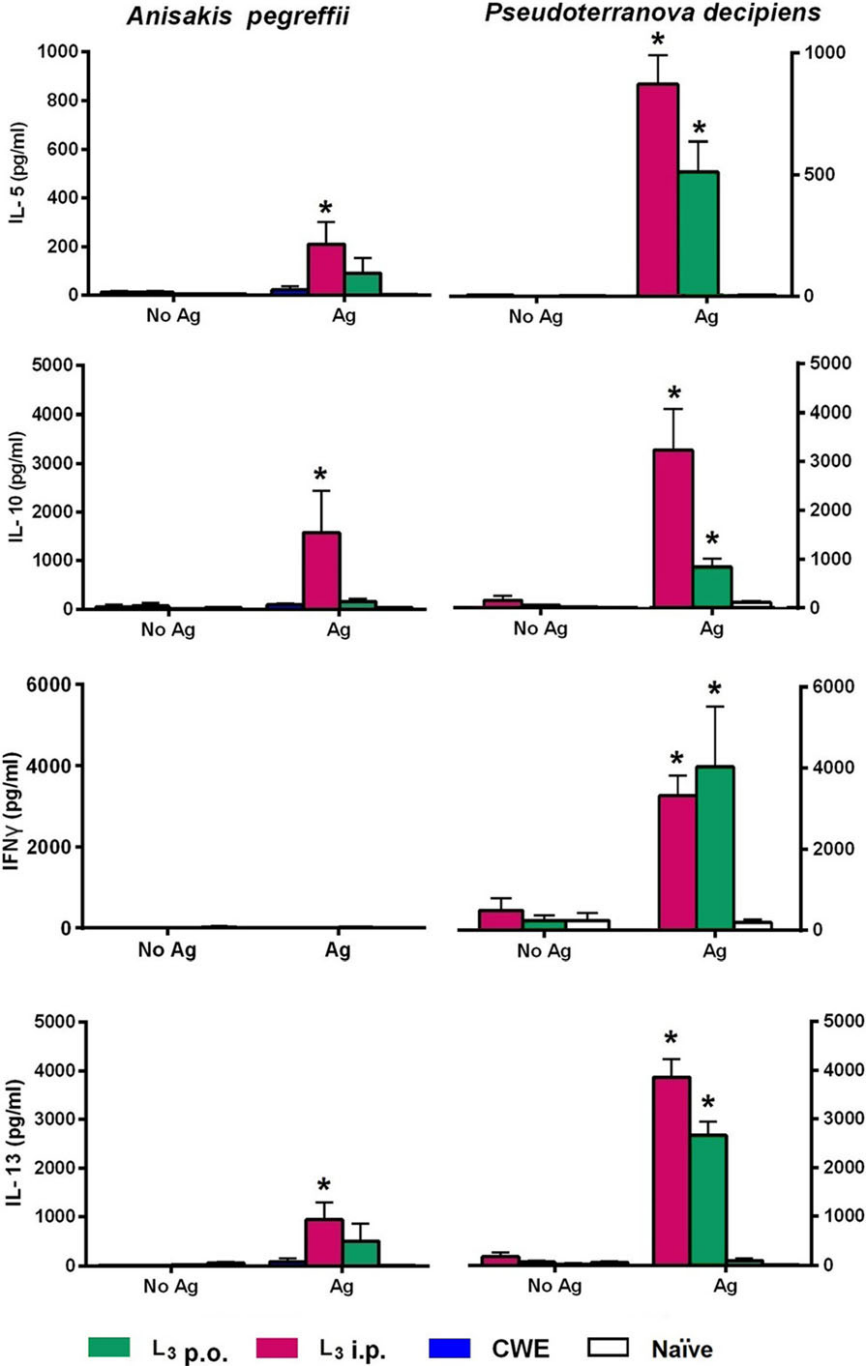
Cytokine production in supernatants of splenocyte cultures stimulated with *Anisakis pegreffii* or *Pseudoterranova decipiens* crude worm antigens (CWE). Splenocytes from BALB/c mice were harvested at week 12. Green histogram: mice orally infected with 2 *A. pegreffii* L3 (L3 p.o.); red histogram: mice intraperitoneally infected with 2 *A. pegreffii* L3 or 2 *P. decipiens* L3 (L3 p.i.)*;* blue histograms: mice orally inoculated with *A. pegreffii* CWE or *P. decipiens* CWE (CWE); open histogram: mice intraperitoneally inoculated with phosphate buffered saline (PBS). *Abbreviations*: No Ag, non-stimulated splenocytes; Ag, splenocytes stimulated with specific CWE. Significances (*P < 0.05) were calculated between experimental and PBS control groups. Each experiment was conducted in triplicate

The antigen-specific stimulation of splenocytes from mice injected i.p. with *A. pegreffii* L3 (group 2Ap) induced significantly higher levels of IL-5, IL-10 and IL-13 than those produced by splenocytes from naïve mice (IL-5 and IL-10*: U*_(0)_ = 2, *Z* = 2.50, *P* = 0.01208; IL-13: *U*_(0)_ = 8, *Z* = 3.000, *P* = 0.027) (Fig. 3). The stimulation of splenocytes from mice injected i.p. with *P. decipiens* L3 (group 1Pd) and mice orally inoculated with CWE from *P. decipiens* (group 2Pd), induced significantly higher levels of IL-5, IL-10, IL-13 and IFN-ɣ than those produced by the splenocytes obtained from naïve mice (*U*_(0)_ = 2, *Z* = 2.50, *P* = 0.01208) (Fig. 3).

**Fig. 4:**
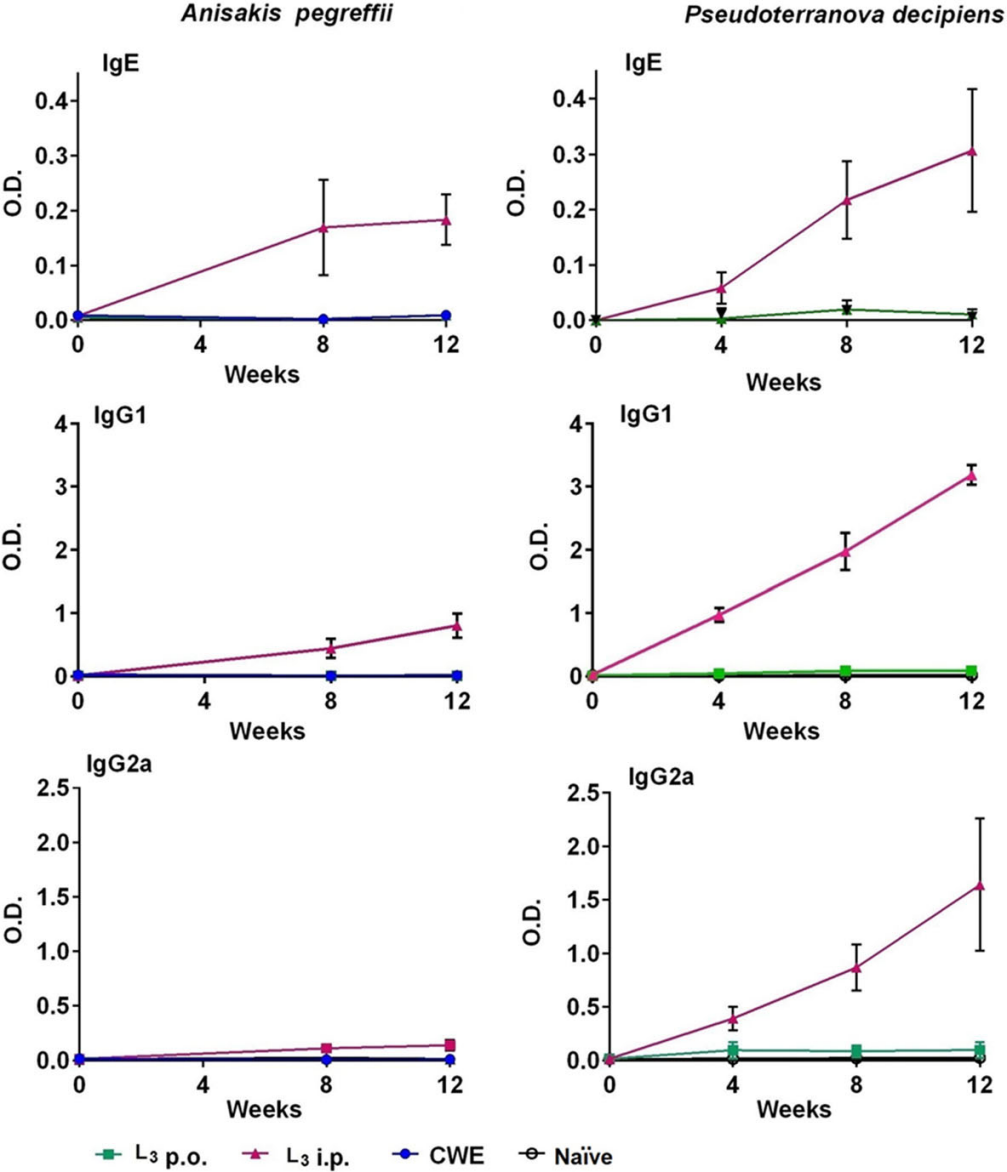
Kinetic of the IgE, IgG1 and IgG2a responses to crude worm extracts (CWE) from *Anisakis pegreffii* or *Pseudoteranova decipiens* in BALB/c mouse sera. Green symbols and lines: mice orally infected with *A. pegreffii* L3 (L3 p.o.); red symbols and lines: mice intraperitoneally (i.p.) infected with *A. pegreffii* L3 or *P. decipiens* L3 (L3 p.i.); blue symbols and lines: mice orally immunized with *A. pegreffii* CWE or *P. decipiens* CWE (CWE); open circles and lines: mice (naïve) intraperitoneally inoculated with phosphate buffered saline (PBS). Symbols represent mean values with SE. Each experiment was conducted in triplicate

The intraperitoneal exposure of BALB/c mice to both *A. pegreffii* L3 and *P. decipiens* L3, induced specific IgE and IgG1 levels in mouse sera from week 8 and 4, respectively, until the last day of observation. Detectable IgG2a levels were found only in the sera from mice i.p. exposed to *P. decipiens* L3 (Fig. 4).

## Discussion

The results suggest that infection with *P. decipiens* is able to sensitize mice to react to subsequent oral challenge with anisakid proteins, as occurs with *A. pegreffii* infection, and that anisakid proteins induce a dominant Th2 response, although *P. decipiens* could also induce a mixed Th1/Th2 pattern.

Several experimental models of *Anisakis* spp. allergic disease were developed in an attempt to elucidate the immunological mechanisms of anisakiasis. These experimental models showed that the *Anisakis* spp. infection predisposes to allergic responses once the host is challenged with *Anisakis* spp. proteins. After challenging, specific IgE and IgG1, and sometimes IgG2a, were detected in serum. Moreover, synthesis of Th2 cytokines was prevalent even if sometimes a mixed Th1/Th2 cell response was found [11, 14, 15, 20–24].

These literature data are comparable with the results of the present study, in which BALB/c mice exhibited allergic symptoms after they were infected i.p. with *A. pegreffii* or *P. decipiens* live L3, reinfected to boost responses, and then orally challenged with 5 mg of proteins. In the developed models, beside symptoms, allergic reactions were further supported by the presence of histamine in the serum samples. However, *P. decipiens* i.p. infection induced mild symptoms and lower histamine release than *A. pegreffii* (Fig. 1).

This immune response could be explained by a lower pathogenic and/or allergenic potential of *P. decipiens* than that of *A. simplex* (*s*.*l.*). Human infections caused by anisakid species as *Pseudoterranova* spp. and *Contracaecum* spp. or by species from a different family (e.g. *Hysterothylacium* spp., Raphidascarididae) are reported to be often mild and transient, causing nausea, cramps, pharyngeal irritation and occasionally allergic symptoms [25, 26]. L3 of these genera can occasionally penetrate the gastric tract to cause acute disease, but on the whole, they are less invasive than L3 of species of the genus *Anisakis* [27, 28]. Regarding allergenic potential, the orogastric infection of mice with live L3 of *Contracaecum* sp. did not elicit serum sensitization [17]. However, no information was published on *Pseudoterranova* spp. experimental infections. So far, 14 different proteins have been identified as *A. simplex* allergens; six of these are recognized by more than 50% of *Anisakis*-allergic patients, and then identified as major allergens [29]. Ani s 7, one of the major *A. simplex* allergens, is not present in, or is antigenically different from *Pseudoterranova* spp. allergens [30]. An immunogenic haemoglobin of 37.6 kDa from *P. decipiens* was isolated and sequenced; it was not immunologically characterized [31] but presents a high degree of homology with Ani s 13, which is a main allergen of *A. simplex* (*s*.*l*.) [32]. Moreover, the *A. pegreffii* haemoglobin is an IgG- and IgE-reacting protein in *Anisakis*-infected mice [33].

Even if there is no detailed information either on other *Pseudoterranova* spp. antigens or allergens, we cannot categorically exclude the presence of such molecules in species of this nematode either own or shared with other members of the same family (Anisakidae) or superfamily (Ascaridida), as for *A. simplex* (*s.l.*) and *Hysterothylacium aduncum* [34].

Besides allergenic proteins, the different anisakid genera could be heterogeneous in terms of presence of immunomodulatory molecules. A different pattern of such molecules could be responsible for the final outcome of the immune response, shifting towards a prevalent Th2 or Th1/Th2 polarization. The immunomodulatory properties of these molecules could also account for the different severity of allergic symptoms elicited by the challenge with the two anisakid extracts.

In the present study, differences in scoring and histamine release among the experimental groups of *A. pegreffii* were observed; whereas, no difference was detected between the experimental groups of *P. decipiens.* For experimental models of anisakid L3 infection, there is a consensus, in which the intraperitoneal infection is the best route of inoculation, since it closely mimics human infection, and it is less traumatic for mice than the oral administration due to the relatively large size of L3. Moreover, by the intraperitoneal route, larvae cannot be discharged, whereas more than 50% of L3 are discharged in orally-infected mice [35]. In our study, no allergic reaction was observed in mice orally infected with *A. pegreffii* L3. On the basis of literature data, we can argue that the orally introduced L3 could have been expelled.

Several reports suggest that only the ingestion of live larvae, which attach themselves to the gastric mucosa, can predispose to allergic reaction in humans [36–38]. In fact, oral challenges with lyophilized L3 or excretory-secretory extracts from L3 to sensitized patients did not induce allergic manifestations [38, 36]. The possibility exists that lyophilized L3 lose their allergenic activities. However, *Anisakis*-associated hypersensitivity cases attributable to the ingestion of cooked, canned, and frozen seafood have also been suggested in countries where the consumption of fish infected with *Anisakis* spp. is highly frequent [2, 39, 40]. Therefore, the allergenic activity of ingested L3 proteins without any treatment, which could alter their allergenic nature, was tested in the present study in mice orally inoculated with 700 μg/mouse of ApCWE or PdCWE. The maximum symptom score observed in both mouse groups was not severe and no histamine release was detected after the oral challenge in comparison with the control mice (Fig. 1). Consequently, the oral inoculation followed by the oral challenge with CWEs did not induce allergic sensitization, which was further confirmed by the absence of an IgE response in those mice (Fig. 4). Since the histamine release was detected in mice sensitized with CWE or in orally injected L3 mice, in which specific immunoglobulin responses were not induced, a direct IgE-independent stimulatory effect on mast cells can be argued.

The cytokine pattern produced by splenocytes from mice inoculated i.p. with *A. pegreffii* or *P. decipiens* L3, which had been activated with anti-CD3/anti-CD28 or with specific antigens, suggests the prominence of a Th2 response (IL-5, IL-10 and IL-13) for the *A. pegreffii* model, and a mixed Th1/Th2 response (IL-5, IL-10, IL-13 and IFN-ɣ) for the *P. decipiens* model (Fig. 2). These results were supported by the mouse humoral immune response (IgE, IgG1 and IgG2a) (Fig. 4). Indeed, *P. decipiens* induce Th2-associated immune response (IgE and IgG1 antibodies, IL-5, IL-10 and IL-13) and also a Th1-like response characterized by high levels of IgG2a antibody and by IFN-γ production, whereas in *A. pegreffii,* Th2 and not Th1-associated immune responses are evident. Similar results were obtained with other murine models of infection with *A. simplex* [11, 23] or *A. pegreffii* [14], in which the *Anisakis* sp. infection induced Th2-biased immune responses with elevated synthesis of IgE and IgG1 and an increased splenocyte production of IL-5, IL-10 and IL-13. The systemic Th2 response can predispose to allergic reactions after an oral challenge with *Anisakis* sp. proteins ending in a type 1 hypersensitivity reaction. Instead, for *Pseudoterranova* spp., no experimental models have been developed so far. In the *P. decipiens* BALB/c animal model described here, a mixed Th1/Th2 pattern was observed, similar to that observed in an *A. simplex* mouse (C3H/He) model, in which mice were sensitized i.p. and then intravenously challenged with *A. simplex* antigens [13]*.*

In the present study, the same mixed Th1/Th2 cytokine patterns (IL-5, IL-10, IL-13 and IFN-ɣ) was produced by splenocytes from mice orally inoculated with 700 μg of ApCWE or PdCWE when stimulated with the specific antigens, but no specific antibody response was found in sera from these mice.

A certain intra-group variability among individual responses was observed along the experiments, despite inbred BALB/c mice were used; this variability is not an uncommon finding in allergy models, since it has been also found in a robust model of cockroach allergen induced asthma-like pulmonary inflammation, in which inbred BALB/c and outbred mice display roughly equivalent levels of overall, intra-strain variability [41].

## Conclusions

The overall results suggest that infection with *P. decipiens* can sensitize mice to react to subsequent oral challenge with anisakid proteins, as described for *A. simplex* (*s*.*s*.) and *A. pegreffii* infections. The results show that anisakid proteins induce a dominant Th2 response, although *P. decipiens* could also induce a mixed type 1/type 2 pattern.

## Abbreviations

1Ap: Mice from *Anisakis pegreffii* group 1
1Pd: Mice from *Pseudoterranova decipiens* group 1
2Ap: Mice from *A. pegreffii* group 2
2-ME: 2-mercaptoethanol
2Pd: Mice from *P. decipiens* group 2
3Ap: Mice from *A. pegreffii* group 3
3Pd: Mice from *P. decipiens* group 3
4Ap: Mice from *A. pegreffii* group 4
Ag: Specific crude worm extract
Ani s: *A. simplex* allergen
ApCWE: *A. pegreffii* crude worm extract
CWE: Crude worm extract
ELISA: Enzyme immunosorbent assay
FBS: Fetal bovine sera
i.p.: Intraperitoneally
IFN: Interferon
Ig: Immunoglobulin
IL: Interleukin
ISS: Istituto Superiore di Sanità
L3: Third-stage larvae
OD: Optical density
p.o.: infected per os
PBS: Phosphate buffered saline
PCR/RLPF: PCR/random amplified polymorphic fragments
PdCWE: *P. decipiens* crude worm extract
SE: Standard error
Th: T-helper
